# African swine fever virus MGF-360-10L is a novel and crucial virulence factor that mediates ubiquitination and degradation of JAK1 by recruiting the E3 ubiquitin ligase HERC5

**DOI:** 10.1128/mbio.00606-23

**Published:** 2023-07-07

**Authors:** Dan Li, Jiangling Peng, Junhuang Wu, Jiamin Yi, Panxue Wu, Xiaolan Qi, Jingjing Ren, Gaochuang Peng, Xianghan Duan, Yi Ru, Huanan Liu, Hong Tian, Haixue Zheng

**Affiliations:** 1 State Key Laboratory for Animal Disease Control and Prevention, College of Veterinary Medicine, Lanzhou University, Lanzhou Veterinary Research Institute, Chinese Academy of Agricultural Sciences, Lanzhou, China; Chunfu Zheng, University of Calgary, Calgary, Canada

**Keywords:** African swine fever virus, MGF-360-10L, HERC5, JAK1, ubiquitination

## Abstract

African swine fever virus (ASFV) causes acute hemorrhagic infectious disease in pigs. The ASFV genome encodes various proteins that enable the virus to escape innate immunity; however, the underlying mechanisms are poorly understood. The present study found that ASFV MGF-360-10L significantly inhibits interferon (IFN)-β-triggered STAT1/2 promoter activation and the production of downstream IFN-stimulated genes (ISGs). ASFV MGF-360-10L deletion (ASFV-Δ10L) replication was impaired compared with the parental ASFV CN/GS/2018 strain, and more ISGs were induced by the ASFV-Δ10L in porcine alveolar macrophages *in vitro*. We found that MGF-360-10L mainly targets JAK1 and mediates its degradation in a dose-dependent manner. Meanwhile, MGF-360-10L also mediates the K48-linked ubiquitination of JAK1 at lysine residues 245 and 269 by recruiting the E3 ubiquitin ligase HERC5 (HECT and RLD domain-containing E3 ubiquitin protein ligase 5). The virulence of ASFV-Δ10L was significantly lower than that of the parental strain *in vivo*, which indicates that MGF-360-10L is a novel virulence factor of ASFV. Our findings elaborate the novel mechanism of MGF-360-10L on the STAT1/2 signaling pathway, expanding our understanding of the inhibition of host innate immunity by ASFV-encoded proteins and providing novel insights that could contribute to the development of African swine fever vaccines.

**IMPORTANCE:**

African swine fever outbreaks remain a concern in some areas. There is no effective drug or commercial vaccine to prevent African swine fever virus (ASFV) infection. In the present study, we found that overexpression of MGF-360-10L strongly inhibited the interferon (IFN)-β-induced STAT1/2 signaling pathway and the production of IFN-stimulated genes (ISGs). Furthermore, we demonstrated that MGF-360-10L mediates the degradation and K48-linked ubiquitination of JAK1 by recruiting the E3 ubiquitin ligase HERC5. The virulence of ASFV with MGF-360-10L deletion was significantly less than parental ASFV CN/GS/2018. Our study identified a new virulence factor and revealed a novel mechanism by which MGF-360-10L inhibits the immune response, thus providing new insights into the vaccination strategies against ASFV.

## INTRODUCTION

African swine fever (ASF) is an acute, severe, and hemorrhagic infectious disease that is caused by African swine fever virus (ASFV), a nucleocytoplasmic large DNA virus that infects domestic pigs and wild boars (1, 2). ASFV infection is characterized by a rapid onset and causes symptoms including high fever, dyspnea, and extensive hemorrhaging of the skin and multiple internal organs in domestic and wild boars (3). ASFV can be divided into 24 genotypes according to the genetic characteristics of the C-terminal sequence variation in the B646L gene, which encodes the major capsid protein p72 (4, 5). These significant differences in genome size are mainly caused by the gain or loss of copies of multigene family (MGF) genes in the variable region at both ends and changes in the number of tandem repeats in the noncoding regions of the ASFV genome (6 - 8). At least five MGFs can be assigned based on the size of the encoded protein: MGF100, MGF110, MGF300, MGF360, and MGF530 (MGF505) (9). The genes in MGF360 and MGF505 can determine the host range and viral virulence, promote the survival of infected cells (10), and to some extent, also affect the host antiviral immune response (11).

The JAK-STAT signaling pathway is crucial for regulating the immune response, and almost all interferons (IFNs) can generate an effective immune response by activating the JAK-STAT signaling pathway (12). IFNs are induced when pattern recognition receptors on the cell surface interact with the nucleic acids of the invading virus (13). IFN-α/β form heterodimers with the IFN-α/β receptor IFNAR, then phosphorylate and activate Janus kinases JAK1 and TYK2. Subsequently, the downstream signal transducer and activator of transcription 1/2 (STAT1/2) are phosphorylated, which forms the IFN-stimulated gene factor 3 (ISGF3) complexes with IFN regulatory factor 9 (IRF9). Activated ISGF3 complexes translocate to the nucleus and specifically bind to IFN-stimulated response elements, thereby activating the transcription of downstream IFN-stimulated genes (ISGs) (14). In addition to the STAT1/2 pathway, type II IFNs can bind to their receptor IFNGR and subsequently activate JAK1 and JAK2, thereby inducing the formation of homodimers of p-STAT1, which enter the nucleus and interact with IFN-γ activation sequence to initiate the expression of the corresponding gene (15).

In a previous study, MGF360/505 deletion mutant was constructed and used to infect macrophages, revealing that MGF360/505 directly or indirectly inhibits the IFN response (16). The deletion of genes that can inhibit IFNs could be used to reduce the virulence of ASFV and thus generate candidate vaccine strains. MGF-360-9L is a major virulence factor that degrades STAT1 and STAT2 to escape host innate immunity (17). ASFV MGF-360-11L can degrade TBK1/IRF7 and inhibit the phosphorylation of TBK1 and IRF3 after transfection with cyclic GMP-AMP synthase (cGAS) and stimulator of interferon gene (STING), thereby inhibiting type I IFN-mediated antiviral activity (18). ASFV MGF-360-12L can competitively inhibit the interaction between importin α and p65 from blocking the nuclear translocation of nuclear factor-κB (NF-κB) (19). However, the role of other ASFV MGF-360 proteins in regulating host innate immunity remains unclear.

In the present study, we characterized the role of MGF-360-10L in inhibiting host innate immunity during ASFV infection. We found that MGF-360-10L can target and degrade JAK1 to reduce the production of ISGs. Mechanistically, we demonstrated that MGF-360-10L promotes the K48-linked ubiquitination of JAK1 by recruiting the E3 ubiquitin ligase HERC5. We also found that the replication ability and virulence of ASFV deleting MGF-360-10L (ASFV-Δ10L) were significantly lower than those of the parental ASFV CN/GS/2018 (wild-type ASFV, ASFV-WT). Our findings clarify the role of the virulence factor MGF-360–10L and its novel mechanism of action on the STAT1/2 signaling pathway and provide a theoretical basis for further research into anti-ASFV drugs and vaccines.

## RESULTS

### Overexpression of MGF-360-10L inhibits STAT1/2 signaling and downstream ISGs

**Fig 1:**
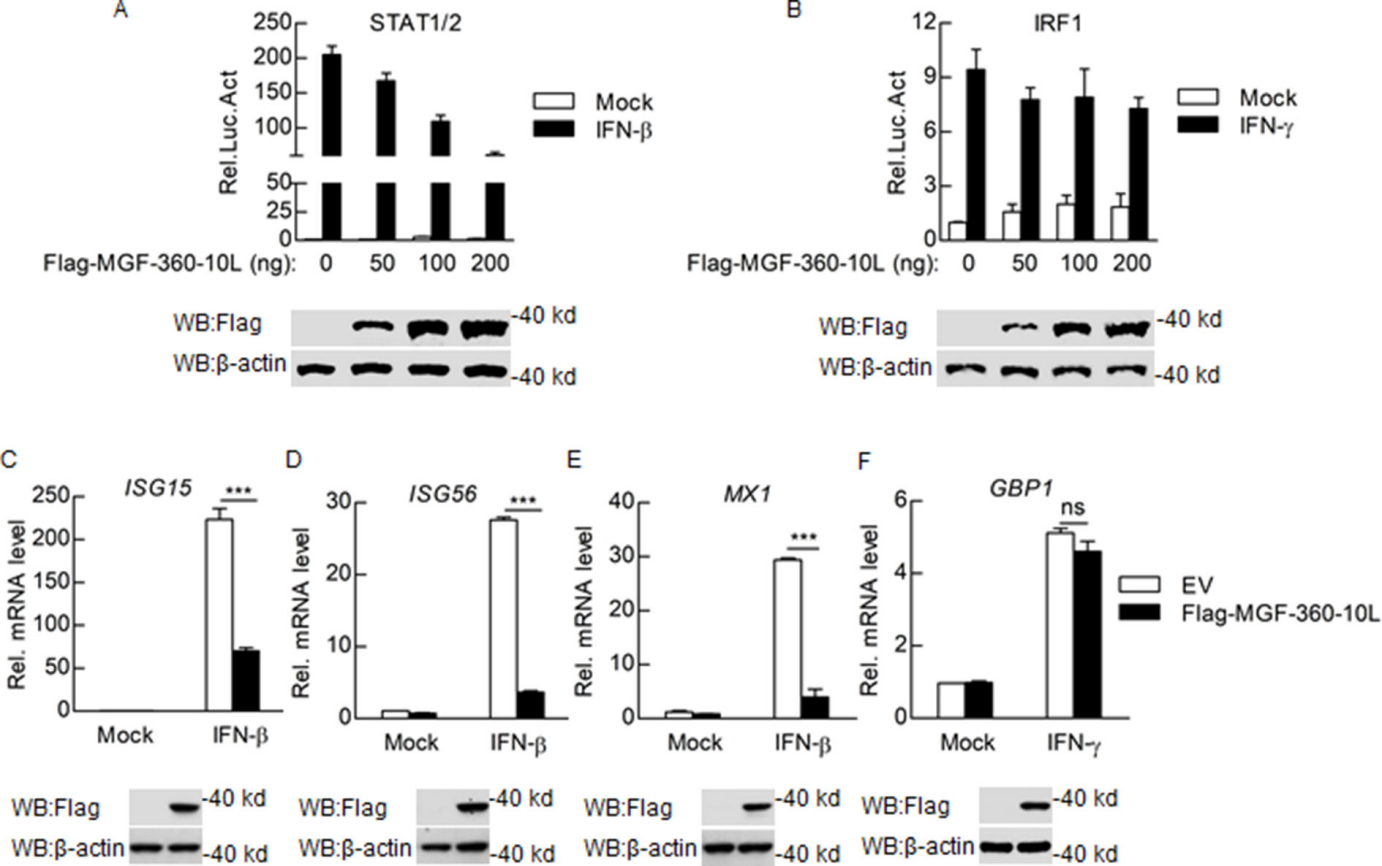
ASFV MGF-360-10L inhibits STAT1/2 signaling. (**A and B**) HEK293T cells in 48-well plates were transfected with Flag-MGF-360-10L overexpression plasmid (0, 0.05, 0.1, or 0.2 µg), along with STAT1/2 (**A**) or IRF1 (**B**) reporter plasmids. After 24 h, cells were stimulated with IFN-β (1,000 U/mL) or IFN-γ (10 ng/mL) for 8 h, and luciferase assays were performed. (**C–E**) HEK293T cells were transfected with empty vector or Flag-MGF-360-10L (1 µg) for 24 h, then stimulated with IFN-β or IFN-γ for 8 h before measuring the transcription levels of *ISG15* (**C**), *ISG56* (**D**), *MX1* (**E**), and *GBP1* (**F**) genes. Data represent the mean ± SD, *n* = 3. *P＜0.05, **P＜0.01, ***P＜0.001.

The JAK-STAT signaling pathway mediates almost all immunoregulatory processes and induces various ISGs and cytokines downstream to exert antiviral effects (20). We had previously determined that MGF-360-9L could inhibit IFN-β-induced activation of the STAT1/2 promoter (17). Meanwhile, we found that ASFV MGF-360-10L also inhibits IFN-β-induced activation of STAT1/2 promoter by luciferase reporter system assays. To further determine the effect of MGF-360-10L on the STAT1/2 signaling pathway, HEK293T cells were transiently transfected with STAT1/2 or IRF1 luciferase reporter plasmids together with plasmids expressing MGF-360-10L. MGF-360-10L could significantly inhibit IFN-β-induced STAT1/2 signaling in a dose-dependent manner but did not affect IFN-γ-induced activation of IRF1 promoter (Fig. 1A and B). We next investigated the function of MGF-360-10L on the levels of IFN-β-triggered downstream ISGs by qPCR. Overexpression of MGF-360-10L could reduce the mRNA levels of downstream *ISG15*, *ISG56*, and *MX1* induced by IFN-β but did not affect the mRNA levels of downstream *GBP1* induced by IFN-γ (Fig. 1C through F). These results indicate that the overexpression of MGF-360-10L inhibits the STAT1/2 signaling pathway and downstream ISGs.

### ASFV MGF-360-10L negatively regulates IFN-β-triggered STAT1/2 signaling

**Fig 2:**
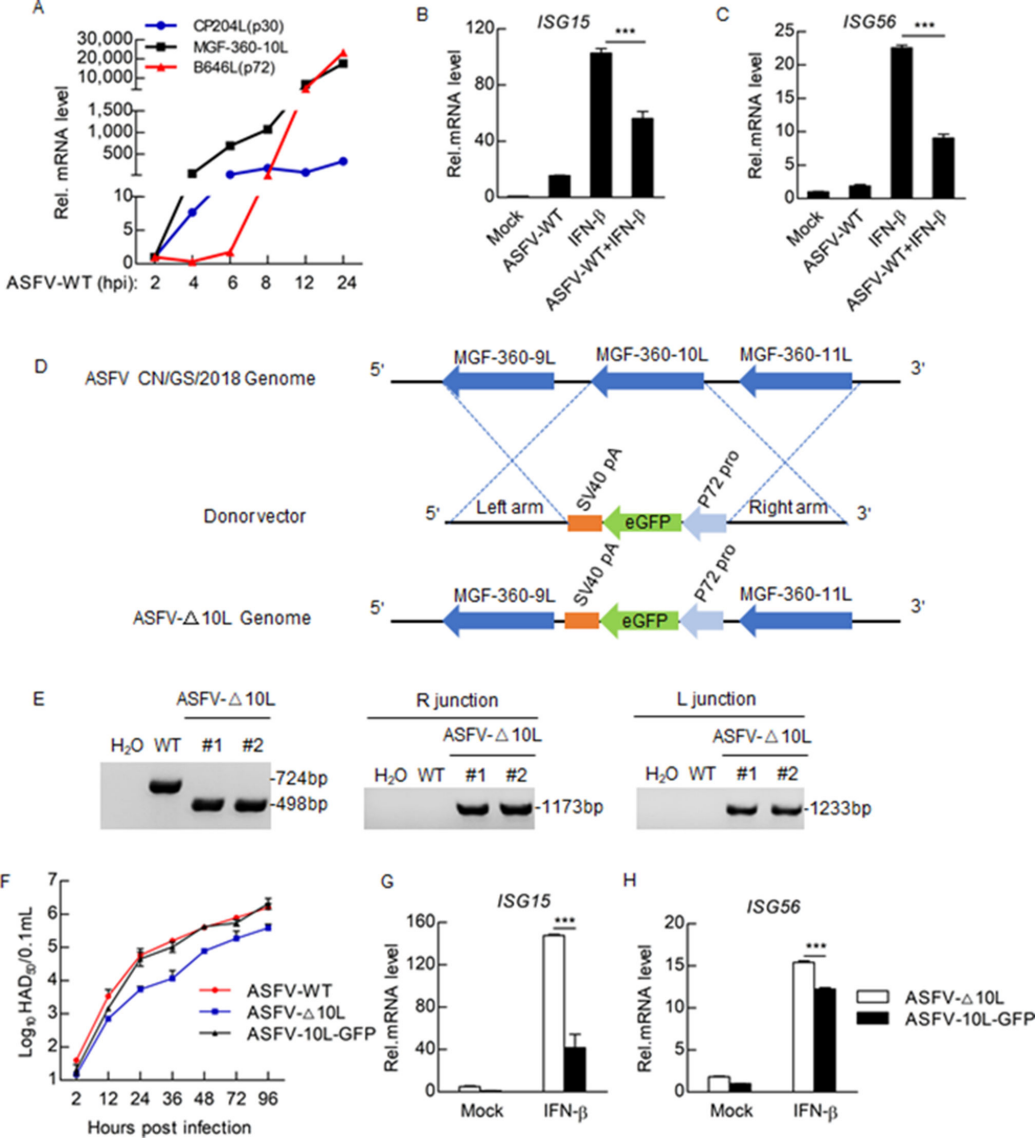
ASFV MGF-360-10L negatively regulates IFN-β-triggered STAT1/2 signaling. (**A**) PAMs were infected with ASFV-WT at the indicated times (2, 4, 6, 8, 12, and 24 h). The mRNA expression levels of MGF-360-10L, CP204L (p30), and B646L (p72) were detected by qPCR. (**B and C**) PAMs were infected with ASFV for 24 h and then treated with IFN-β (0.7 µg/mL) for 4 h before measuring the transcription levels of *ISG15* (**B**) and *ISG56* (**C**). (**D**) Diagram indicating the position of the MGF-360-10L open reading frame in the ASFV CN/GS/2018 genome. (**E**) The absence of parental CN/GS/2018 was confirmed using PCR. (**F**) PAMs were infected with the parental ASFV-WT, ASFV-10L-GFP, or ASFV-Δ10L (MOI: 0.01) strains for the indicated time before HAD_50_ was detected. (**G and H**) PAMs were infected with ASFV-10L-GFP or ASFV-Δ10L for 24 h and then treated with IFN-β (0.7 µg/mL) for 4 h before *ISG15* (**G**) and *ISG56* (**H**) were detected by qPCR. Data represent the mean ± SD, *n* = 3. *P＜0.05, **P＜0.01, ***P＜0.001.

To determine the effect of ASFV on IFN-β-triggered STAT1/2 signaling, we first needed to understand the biological properties of MGF-360-10L. To determine the kinetics of MGF-360-10L transcription, total RNA was extracted from porcine alveolar macrophages (PAMs) infected with ASFV-WT, and the mRNA levels were determined by qPCR. MGF-360-10L mRNA was expressed in the early phase of infection simultaneously with p30 (Fig. 2A). Meanwhile, PAMs infected with ASFV-WT and treated with IFN-β had significantly reduced the mRNA levels of *ISG15* and *ISG56* (Fig. 2B and C). These results indicate that ASFV-WT negatively regulates IFN-β-triggered STAT1/2 signaling.

To verify the effect of endogenous ASFV MGF-360-10L on STAT1/2 signaling, we constructed ASFV-Δ10L and ASFV-10L-GFP [a 3′-terminally enhanced green fluorescent protein (eGFP)-tagged virus] by homologous recombination. The MGF-360-10L gene was either replaced or the 3′-terminus was added with eGFP using a cassette containing the eGFP gene under the control of the ASFV p72 promoter (Fig. 2D; Fig. S1A). The recombinant viruses were purified via 11 rounds of limited dilution by selecting eGFP-positive cells (Fig. S1B). The purity of ASFV-Δ10L and ASFV-10L-GFP was confirmed by PCR (Fig. 2E; Fig. S1C).

A previous publication demonstrated that the adsorption of red blood cells on the surface of ASFV-infected macrophages is a unique phenomenon which allows for the determination of viral infectious titers by hemadsorption (HAD) (21 - 23). To explore the effect of MGF-360-10L on ASFV replication *in vitro*, PAM cells were infected with ASFV-WT, ASFV-Δ10L, or ASFV-10L-GFP, and viral titers were detected using the 50% HAD (HAD_50_). Compared with the ASFV-WT- and ASFV-Δ10L-GFP-infected cells, ASFV-Δ10L reduced the replication of ASFV in PAMs (Fig. 2F; Fig. S1D). ASFV-Δ10L also significantly increased the expression of *ISG15* and *ISG56* compared to ASFV-10L-GFP or ASFV-WT (Fig. 2G and H; Fig. S1E and F). These results suggest that ASFV MGF-360-10L negatively regulates IFN-β-triggered STAT1/2 signaling.

### MGF-360-10L targets JAK1 on STAT1/2 signaling pathway

**Fig 3:**
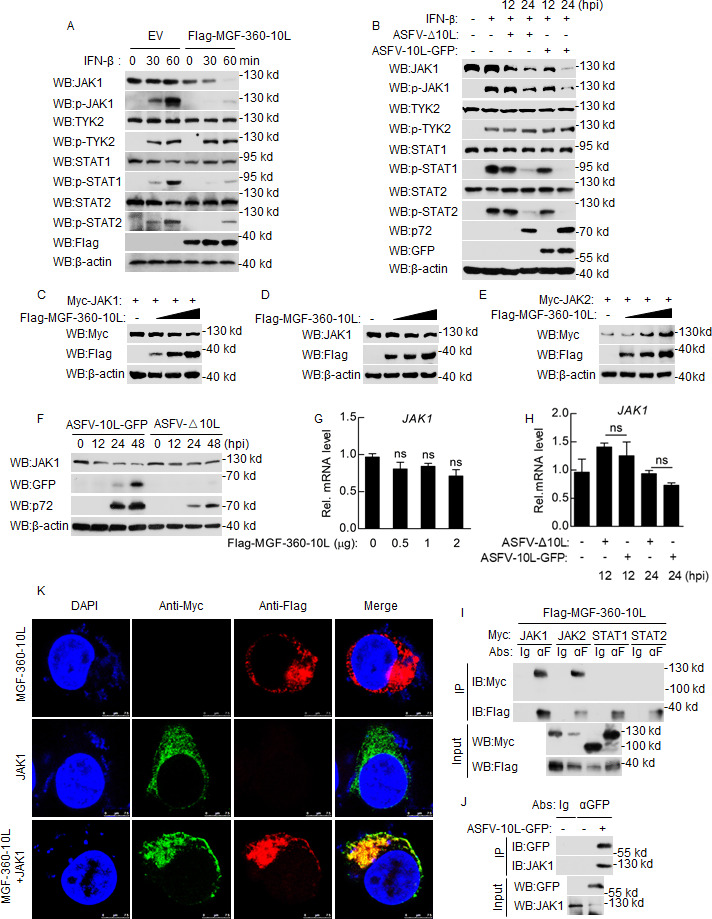
MGF-360-10L targets and colocalizes with JAK1. (**A**) HEK293T cells were transfected with Flag-MGF-360-10L expression plasmid or empty vector for 24 h, then treated with IFN-β (1,000 U/mL) for the indicated time. Cell lysates were analyzed by Western blotting with the indicated antibodies. (**B**) PAMs were infected with ASFV-10L-GFP or ASFV-Δ10L (MOI: 0.01) for the indicated time, then treated with IFN-β (0.7 µg/mL) before Western blotting. (**C–E**) HEK293T cells were transfected with Myc-JAK1 (1 µg) (**C**), Myc-JAK2 (1 µg) (**E**), and Flag-MGF-360-10L (0, 0.5, 1, and 2.0 µg) (**D**) for 24 h, and cell lysates were then analyzed by Western blotting with the indicated antibodies. (**F**) PAMs were infected with ASFV-10L-GFP or ASFV-Δ10L, cell lysates were analyzed by Western blotting. (**G and H**) Total RNA in HEK293T (**G**) and PAMs (**H**) were extracted to detect the mRNA levels of JAK1. (**I**) HEK293T cells were transfected with Myc-JAK1, Myc-JAK2, Myc-STAT1, Myc-STAT2, and Flag-MGF-360-10L. Co-immunoprecipitation and Western blotting analyses were performed using the indicated antibodies. (**J**) PAMs were infected with ASFV-10L-GFP for 24 h, and co-immunoprecipitation and Western blotting analyses were performed with the indicated antibodies. (**K**) HEK293T cells were co-transfected with empty vector or Flag-MGF-360-10L and Myc-JAK1 plasmids for 24 h. The subcellular localization of Flag-MGF-360-10L and Myc-JAK1 in the cells were observed using a confocal microscope. Data represent the mean ± SD, *n* = 3. *P＜0.05, **P＜0.01, ***P＜0.001. DAPI, 4′, 6′-diamidino-2-phenylindole.

Various components are involved in IFN-β-triggered STAT1/2 signaling pathways, included JAK1, TYK2, STAT1, and STAT2 (24). To investigate the target molecules of MGF-360-10L, HEK293T cells were overexpressed empty vector or MGF-360-10L plasmids and stimulated with IFN-β at different times. MGF-360-10L expression reduces JAK1 levels, indicating that MGF-360-10L inhibits upstream molecules of the STAT1/2 signaling pathway, thereby significantly inhibiting STAT1 and STAT2 phosphorylation upon IFN-β treatment (Fig. 3A). To confirm these results further, PAMs were infected with ASFV-Δ10L or ASFV-10L-GFP for 12 and 24 h, then treated with IFN-β. The degradation of JAK1 was attenuated in PAMs infected with ASFV-Δ10L compared to that of cells infected with ASFV-10L-GFP (Fig. 3B). In addition, MGF-360-10L suppresses JAK1 expression in a dose-dependent manner in HEK293 cells (Fig. 3C and D). Meanwhile, MGF-360-10L could promote the expression of JAK2 (Fig. 3E), which is also why MGF-360-10L has no significant effect on the IFN-γ-induced activation of the IRF1 promoter. PAMs were also infected with ASFV-Δ10L and ASFV-10L-GFP to detect their effect on JAK1 expression at the protein level. ASFV-10L-GFP inhibits the expression of JAK1 more strongly than ASFV-Δ10L (Fig. 3F). Conversely, the mRNA levels of JAK1 were not altered by MGF-360-10L (Fig. 3G and H). These results suggest that MGF-360-10L inhibits the STAT1/2 signaling pathway by targeting JAK1 at the protein level.

To investigate the molecular mechanisms underlying the MGF-360-10L-mediated degradation of JAK1, we first clarified the association between MGF-360-10L and JAK1. HEK293T cells were transiently transfected with MGF-360-10L and JAK1, JAK2, STAT1, and STAT2 expression plasmids and used to verify that MGF-360-10L interacts with JAK1/2 in HEK293T cells (Fig. 3I). The interaction between MGF-360-10L and JAK1 was also confirmed in PAMs by co-immunoprecipitation (Fig. 3J). Next, HEK293T cells were co-transfected with plasmids expressing MGF-360-10L and JAK1, and confocal microscopy was used to reveal that MGF-360-10L could colocalize with JAK1 (Fig. 3K). Collectively, these results confirm that MGF-360-10L targets interact with JAK1.

### K245 and K269 residues of JAK1 are critical for MGF-360-10L-mediated ubiquitination and degradation

**Fig 4:**
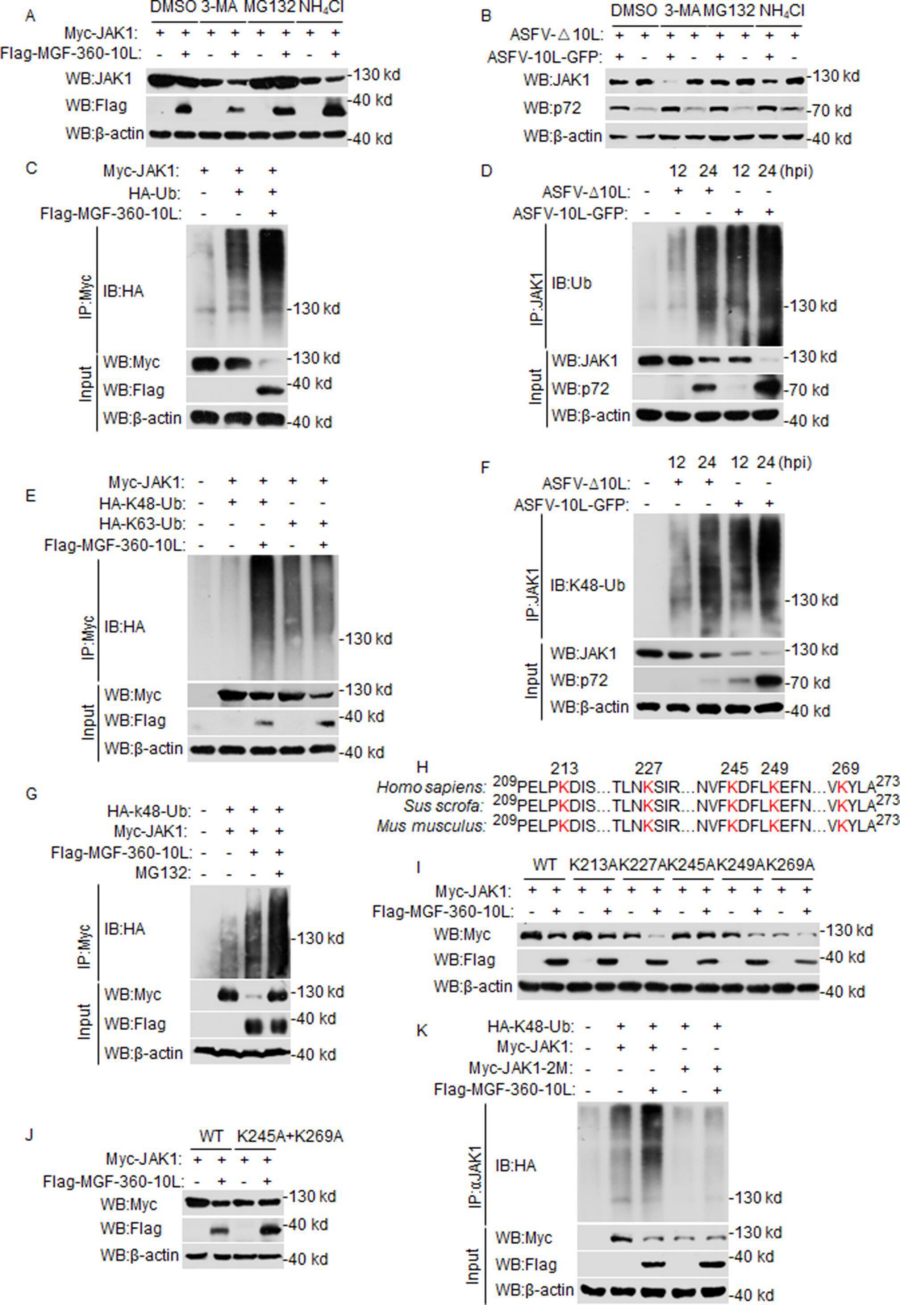
MGF-360-10L promotes K48-linked polyubiquitination of JAK1. (**A**) HEK293T cells were transfected with Flag-MGF-360-10L and Myc-JAK1 for 18 h, then treated with MG132 (100 µM), NH_4_Cl (25 mM), or 3-MA (500 ng/mL) for 6 h before Western blotting. (**B**) PAMs were infected with ASFV-10L-GFP or ASFV-Δ10L (MOI: 0.01) for 24 h, then treated with the indicated inhibitors for 6 h before Western blotting. (**C and E**) HEK293T cells were transfected with Myc-JAK1 (5 µg), HA-Ub (2 µg), HA-K48 (2 µg), HA-K63 (2 µg), or Flag-MGF-360-10L (10 µg) for 24 h, Co-immunoprecipitation and Western blotting analyses were performed using the indicated antibodies. (**D and F**) PAMs were infected with ASFV-10L-GFP or ASFV-Δ10L (MOI: 0.1) for the indicated times, and co-immunoprecipitation and Western blotting analyses were performed using the indicated antibodies. (**G**) HEK293T cells were transfected with Myc-JAK1 (5 µg), HA-K48 (2 µg), and Flag-MGF-360-10L (10 µg) for 18 h, then treated with MG132. Co-immunoprecipitation and Western blotting analyses were performed using the indicated antibodies. (**H**) Highly conserved lysine (**K**) residues on JAK1 in different species according to a multiple sequence alignment. (**I and J**) HEK293T cells were transfected with Flag-MGF-360–10L, Myc-JAK1, or Myc-JAK1 mutants and then cell lysates were analyzed by Western blotting. (**K**) HEK293T cells were transfected with Flag-MGF-360-10L, HA-K48, Myc-JAK1, or Myc-JAK1 mutant plasmids, co-immunoprecipitation, and Western blotting analyses were performed using the indicated antibodies.

To investigate the mechanisms underlying the role of MGF-360-10L in the stability of JAK1, we treated cells with various inhibitors to identify the protein degradation pathways. HEK293T cells were transfected with the MGF-360-10L expression plasmid and treated with different inhibitors. In addition, PAMs were infected with ASFV-Δ10L or ASFV-10L-GFP to determine the endogenous JAK1 degradation pathways. The degradation of JAK1 was completely inhibited by the proteasome inhibitor MG132 but not by the lysosome inhibitor ammonium chloride (NH_4_Cl) or the autophagosome inhibitor 3-MA (Fig. 4A). ASFV-10L-GFP-mediated JAK1 degradation was also completely inhibited by MG132 (Fig. 4B).

The ubiquitin-proteasome system is a proteolytic mechanism that degrades the protein substrate bound to the translational ubiquitin polymer through the enzymatic action of ubiquitin ligase in a process called ubiquitination (25). Since MGF-360-10L degrades JAK1 via the proteasome pathway, we examined whether MGF-360-10L expression increases JAK1 ubiquitination. MGF-360-10L overexpressed in HEK293T cells, or ASFV-10L-GFP infected in PAMs could increase JAK1 ubiquitination, but ASFV-Δ10L did not significantly increase JAK1 ubiquitination compared with ASFV-10L-GFP (Fig. 4C and D). Since K48- and K63-linked ubiquitination are the most common types of ubiquitination (26), we next determined whether MGF-360-10L catalyzed K48- or K63-linked ubiquitination of JAK1. When HEK293T cells were co-transfected with MGF-360-10L and JAK1, or PAMs were infected with ASFV-10L-GFP and ASFV-Δ10L, MGF-360-10L catalyzed K48- rather than K63-ubiquitinated JAK1 (Fig. 4E and F). In addition, the K48 ubiquitination of JAK1 was increased after treating with MG132 (Fig. 4G). These results indicate that MGF-360-10L increases JAK1 ubiquitination and promotes K48-linked polyubiquitination of JAK1.

Previous studies have shown that the N-terminus of JAK1 contains a ubiquitin-like domain (27). We further analyzed the lysine residues in JAK1 using the Protein Lysine Modifications Database. We identified five ubiquitinated lysine residues in JAK1 (K213, K227, K245, K249, and K269) conserved in *Homo sapiens*, *Sus scrofa*, and *Mus musculus* (Fig. 4H). To investigate the specific residues of JAK1 degradation by MGF-360-10L, JAK1 mutants (K213A, K227A, K245A, K249A, and K269A) were generated, in which lysine residues were replaced with alanine residues at positions 213, 227, 245, 249, and 269, respectively. HEK293T cells were co-transfected with these JAK1 mutants and MGF-360-10L expression plasmid. Mutations at either K245 or K269 significantly attenuated the inhibition of JAK1 (Fig. 4I). Furthermore, double mutations (2M) of JAK1 at K245 and K269 largely abolished the inhibition of JAK1 (Fig. 4J). We further examined the effect of 2M on JAK1 ubiquitination and found that JAK1-2M ubiquitination was significantly reduced compared to the wild-type JAK1 (Fig. 4K). Based on these results, MGF-360-10L primarily targets K245 and K269 of JAK1.

### MGF-360-10L mediates JAK1 degradation via the E3 ubiquitin ligase HERC5

**Fig 5:**
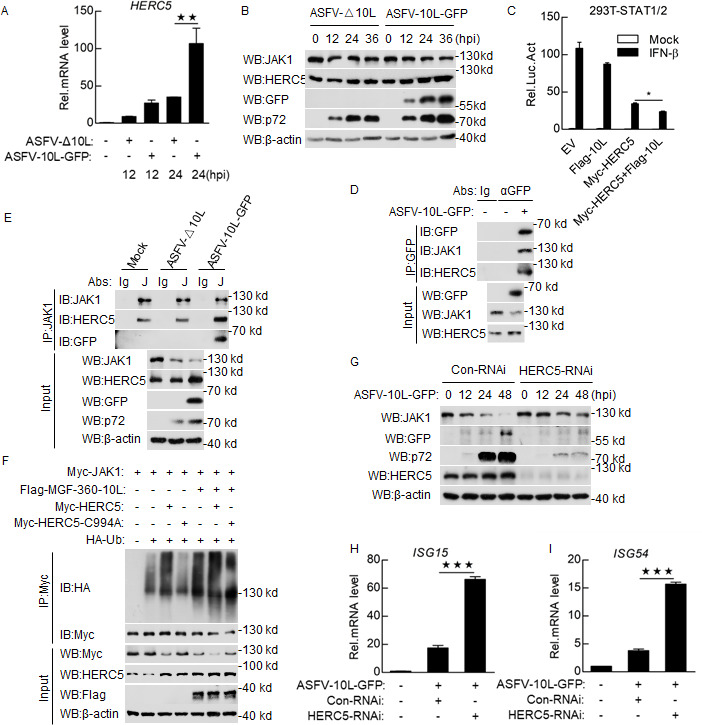
MGF-360-10L mediates JAK1 degradation via E3 ubiquitin ligase HERC5. (**A**) Total RNA was extracted from infected or uninfected PAMs, and the transcription levels of HERC5 were determined by qPCR. (**B**) PAMs were infected with ASFV-10L-GFP or ASFV-Δ10L (MOI: 0.1) for 24 h. Cell lysates were analyzed by Western blotting. (**C**) HEK293T cells in 48-well plates were transfected with Flag-MGF-360-10L or Myc-HERC5 along with STAT1/2 reporter plasmids, and luciferase assays were then performed. (**D and E**) PAMs were infected with ASFV-10L-GFP or ASFV-Δ10L (MOI: 0.1) for 24 h. Co-immunoprecipitation and Western blotting analyses were performed. (**F**) HEK293T cells were transfected with Flag-MGF-360-10L, Myc-HERC5, HA-Ub, and Myc-JAK1 or Myc-HERC5 mutant plasmids. Co-immunoprecipitation and Western blotting analyses were performed. (**G–I**) PAMs were transfected with Con-RNAi or HERC5-RNAi (1.0 µg/mL) for 48 h, then infected with ASFV-10L-GFP (MOI: 0.05) for the indicated times. Cell lysates were analyzed by Western blotting (**G**), or total RNA was extracted to detect the transcription levels of *ISG15* (**H**) and *ISG56* (**I**). Data represent the mean ± SD, *n* = 3. *P＜0.05, **P＜0.01, ***P＜0.001.

Since our results suggest that MGF-360-10L can ubiquitinate JAK1, we next examined which E3 ubiquitin ligase mediates MGF-360-10L degradation of JAK1. PAMs were infected with ASFV-10L-GFP or ASFV-Δ10L to detect the transcriptomic changes over time using transcriptome sequencing (RNA-seq). The E3 ubiquitin ligase HERC5 was significantly upregulated upon ASFV-10L-GFP infection at the mRNA and protein level (Fig. 5A and B; Fig. S2A). To verify the effect of HERC5 on IFN-β-induced STAT1/2 pathway, HEK293T cells were co-transfected with MGF-360-10L and HERC5 expression plasmids, and STAT1/2 luciferase reporter plasmids. Overexpression of HERC5 reduced IFN-β-induced activation of the STAT1/2 promoter, and overexpression of both MGF-360-10L and HERC5 further inhibited STAT1/2 promoter activation (Fig. 5C). Collectively, these results suggest that HERC5 is involved in the degradation of JAK1 by MGF-360-10L.

Previous studies have reported that HERC5 possesses ubiquitin ligase activity (28, 29). Therefore, we focused on HERC5 to examine its potential role in regulating JAK1 degradation. Endogenous co-immunoprecipitation experiments indicated that MGF-360-10L interacted with HERC5 and JAK1 in ASFV-10L-GFP-infected cells (Fig. 5D). In addition, co-immunoprecipitation and Western blotting indicated that MGF-360-10L could promote the expression of HERC5 (Fig. 5E). To understand the effect of MGF-360-10L on HERC5, HEK293T cells were transfected with different doses of Flag-MGF-360-10L. We found that MGF-360-10L could not only mediate the degradation of JAK1 but also promote the expression of HERC5 (Fig. S2B). HEK293T cells were also transfected with the HERC5 expression plasmid at different doses, revealing that HERC5 promotes endogenous JAK1 ubiquitination in a dose-dependent manner (Fig. S2C). Similar to previous studies, JAK1 ubiquitination did not significantly increase with JAK1 point mutations, even after the addition of the E3 ubiquitin ligase HERC5 (Fig. S2D). These results confirm the interactions between HERC5, MGF-360-10L, and JAK1 and suggest that HERC5 has a synergistic effect on MGF-360-10L.

Previous studies have indicated that the conserved cysteine residue C994 of the HECT domain is essential for HERC5 E3 ligase activity and that a targeted substitution of this cysteine with alanine completely abrogates E3 protein ligase activity (30 - 32). Therefore, we constructed HERC5 containing a cysteine to alanine point mutation at residue 994 (HERC5-C994A) and co-transfected MGF-360-10L, JAK1, and HERC5/HERC5-C994A into HEK293T cells. As expected, HERC5-C994A reduced the degradation of JAK1 compared to HERC5 (Fig. S2E). Next, we examined the effects of HERC5 and HERC5-C994A on JAK1 ubiquitination by co-immunoprecipitation and Western blotting, respectively. As shown in Fig. 5F, HERC5 increased the ubiquitination of JAK1 compared to HERC5-C994A (lines 3 and 4), and ubiquitination was more pronounced after the expression of MGF-360-10L (lines 6 and 7). Next, to explore the effect of HERC5 on JAK1, we synthesized small interfering RNAs (siRNA) targeting HERC5 (Fig. S2F) and found that the expression of JAK1 increased in HERC5-knockdown PAMs infected with ASFV-10L-GFP (Fig. 5G). In addition, the number of genomic copies and TCID_50_ after ASFV-10L-GFP infection revealed that the replication of ASFV was significantly weakened after HERC5 knockdown compared to the control siRNA groups (Fig. S2G and H). Importantly, compared to the control siRNA groups, ASFV-10L-GFP-induced transcription of *ISG15* and *ISG56* was upregulated in the HERC5-knockdown groups (Fig. 5H and I). These results indicate that MGF-360-10L recruits HERC5 to improve the degradation of JAK1.

### Evaluate ASFV-Δ10L virulence in swine

**Fig 6:**
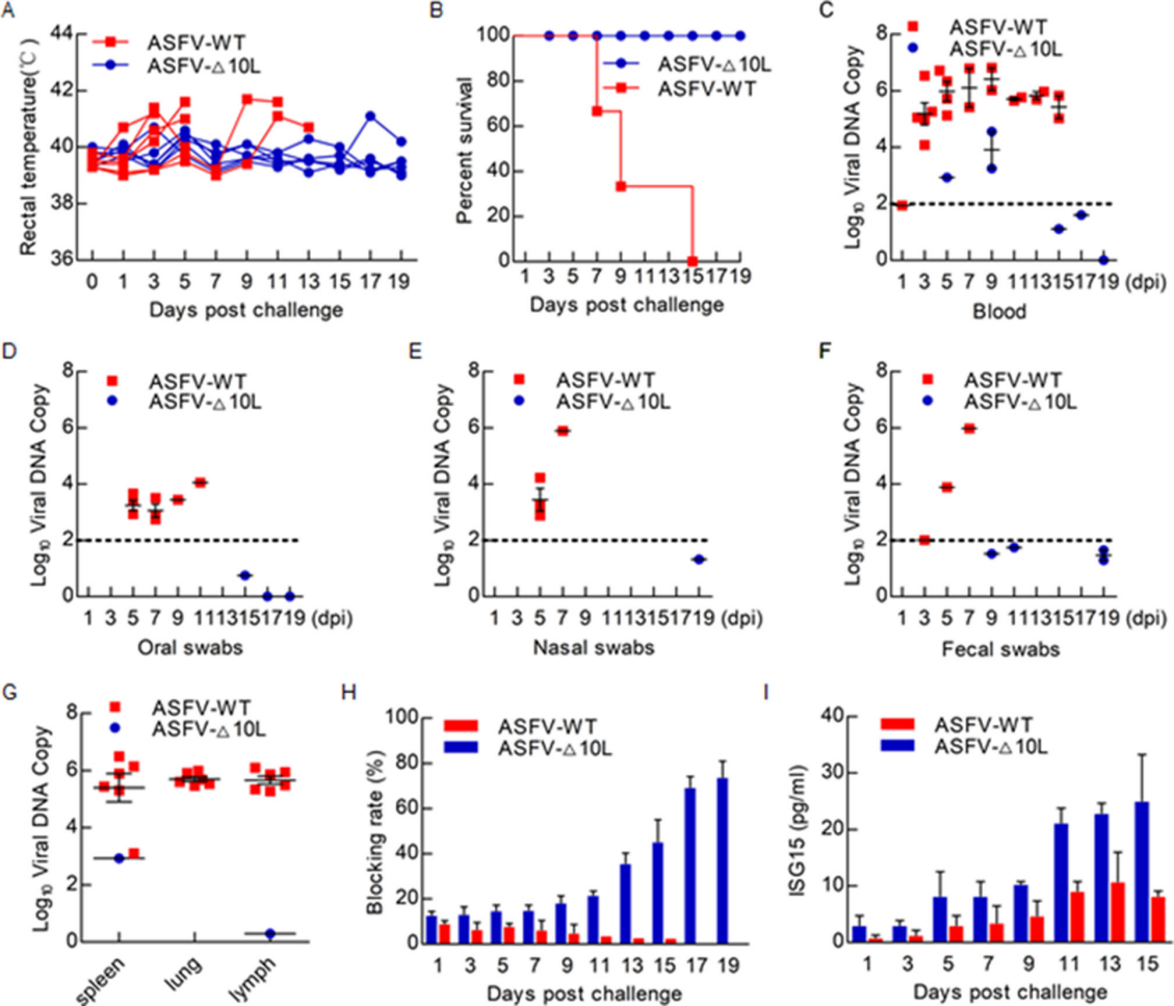
Evaluate ASFV-Δ10L virulence in swine. (**A–H**) Pigs were inoculated intramuscularly with either 10 HAD_50_ of ASFV-Δ10L (*n* = 6) or ASFV-WT (*n* = 6). The pigs were monitored daily for 19 days. (**A**) Daily temperature changes. (**B**) Survival rates. (**C–G**) Number of viral DNA copies in the blood (**C**), swabs (**D–F**), and tissues (**G**) collected at the indicated days post-challenge. (**H and I**) p30 Antibody level (**H**) and ISG15 protein level (**I**) were detected by enzyme-linked Immunosorbent assay (ELISA).

To evaluate the virulence of ASFV-Δ10L, pigs were inoculated intramuscularly with 10 HAD_50_ of the ASFV-WT or ASFV-Δ10L. As expected, pigs infected with ASFV-WT exhibited increased body temperature within 3–4 days after injection, accompanied by diarrhea, anorexia, and skin erythema, and all died within 15 dpi. In contrast, pigs inoculated with the ASFV-Δ10L virus displayed a moderate body temperature increase, and two of the pigs had mild clinical symptoms, but all survived until 19 dpi (Fig. 6A and B). Pigs infected with the ASFV-Δ10L virus had much lower viral loads in the blood compared to pigs infected with ASFV-WT (Fig. 6C). Highly virulent ASFV can result in the rapid death of most infected animals and persistent infection for viral perpetuation and transmission in domestic pigs (33, 34). To investigate viral shedding in pigs infected with ASFV-Δ10L or ASFV-WT, viral loads were determined in oral, nasal, and fecal swabs. All the animals infected with ASFV-Δ10L had significantly lower virus loads in swabs than that of ASFV-WT (Fig. 6D–F). Similar to the viral titers in the blood and swabs, lower viral copies were found in the spleen, lung, and lymph samples from ASFV-Δ10L-infected pigs (Fig. 6G). The surviving pigs infected with ASFV-Δ10L displayed a gradual increase in p30 antibody at the late stages of infection, while ASFV-WT-infected pigs did not induce a p30 antibody response (Fig. 6H). In addition, we explored whether ASFV-Δ10L challenge increases the ISG15 production (Fig. 6I). ISG15 was increased in ASFV-Δ10L challenge groups. These results indicate that MGF-360-10L is a major virulence gene, and ASFV-Δ10L was significantly attenuated in pigs.

### ASFV-Δ10L causes fewer lesions than the parental virus

**Fig 7:**
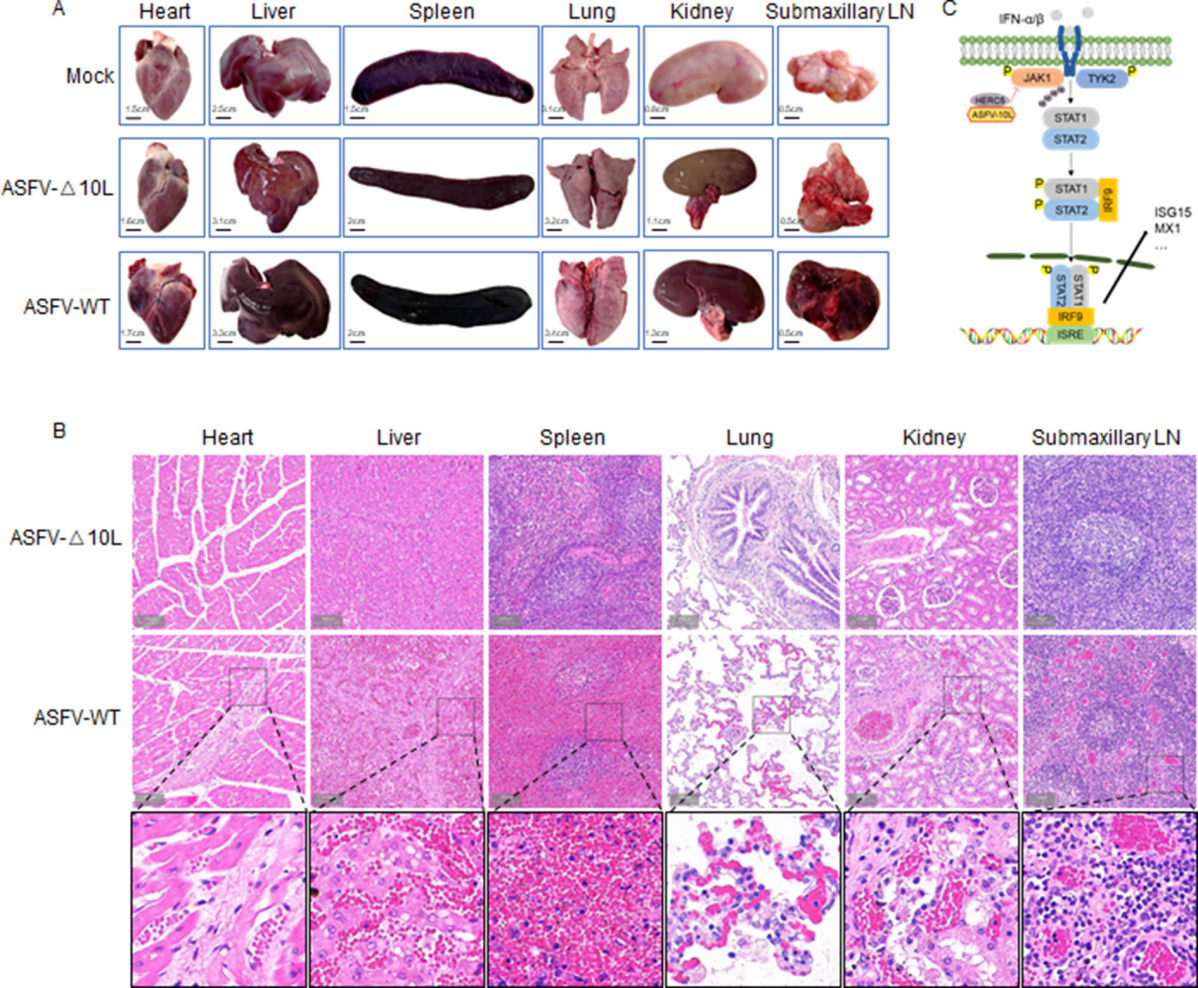
The lesions caused by ASFV-Δ10L were significantly lower than that of the parental ASFV-WT strain. (**A and B**) The pathological (**A**) and histopathological changes (**B**) in different tissues after ASFV-WT or ASFV-Δ10L infection. (**C**) Schematic illustration of the mechanism of inhibition by MGF-360-10L-mediated STAT1/2 signaling.

Pathological changes caused by ASFV infection include enlargement and hemorrhagic necrosis of multiple organs, especially splenomegaly and lymph node hemorrhage (35, 36). We dissected the experimental animals to compare the pathological changes after infection with ASFV-Δ10L or ASFV-WT. ASFV-WT infection resulted in lesions on the expected organs, especially splenomegaly, hemorrhagic lymph nodes, and pulmonary congestion. At the same time, the pathological symptoms were less prevalent in animals infected with ASFV-Δ10L (Fig. 7A). Furthermore, we observed diffuse hemorrhagic spots, lymphocyte necrosis, and nuclear fragmentation in the cortex and medulla of lymph nodes in animals infected with ASFV-WT. The alveolar cavity was dilated and congested, with severe hemorrhage observed in the spleen and kidneys. Importantly, these above pathological changes were weakened or disappeared after infection with ASFV-Δ10L (Fig. 7B). Taken together, these results indicate that the disease severity caused by ASFV-Δ10L was less than that caused by parental ASFV-WT.

## DISCUSSION

In this study, we examined the role of ASFV MGF-360-10L in regulating the host innate immune response. The replication and virulence of ASFV-Δ10L decreased significantly compared to ASFV-WT, verifying that MGF-360-10L is an essential virulence gene. We further confirmed that MGF-360-10L targets JAK1, significantly increasing its ubiquitination in HEK293T cells and PAMs, leading to JAK1 degradation via the proteasome pathway. Next, we screened E3 ubiquitin ligases using RNA-seq and found that MGF-360-10L further promotes the ubiquitination of JAK1 by recruiting HERC5, thereby inhibiting the host innate immune response.

JAK1 plays an important role in type I/II IFN-stimulated cellular signal transduction (37). There are seven conserved JAK homologous domains that contain an N-terminal FERM domain, followed by an Src homologous 2 domain, a pseudokinase domain, and a C-terminal tyrosine kinase domain (38, 39). Influenza A virus polymerase protein PB2 targets mammalian JAK1 at K859 and K860 for ubiquitination and degradation (40). This study found that residues K245 and K269 of JAK1 were critical for ASFV MGF-360-10L-mediated ubiquitination and degradation. The E3 ubiquitin ligase STIP1 homology and U-box containing protein 1 mediates ubiquitination-dependent proteasomal degradation of the IFNγ-R1/JAK1 complex through IFNγ-R1^K285^ and JAK1^K249^ (41). Receptor activator of NF-κB ligand stimulation regulates JAK1 expression through ubiquitin-mediated proteasome degradation (42, 43). In addition, ring finger protein 125 (RNF125) binds to and ubiquitinates JAK1, whereas JAK1 mRNA expression is not affected by RNF125 (44, 45). Although the above studies identified proteins capable of ubiquitinating JAK1, the specific mechanisms remain unclear. The evolutionarily ancient restriction factor HERC5 plays an important role in mammalian innate immunity by interacting with viral proteins to disrupt viral replication (31, 46). In this study, we demonstrated that HERC5 is a novel E3 ubiquitin ligase for JAK1, and HERC5 promotes the ubiquitination of JAK1 via MGF-360-10L. Furthermore, the synergistic effects were reduced by mutation of the ligase active site HERC5-C994A.

There is no effective vaccine or drug for ASFV, and the only efficacious experimental vaccine candidates for ASFV are live attenuated strains generated by deleting specific virulence-associated ASFV genes (47). Deletion of I177L (ASFV-G-ΔI177L) in a highly virulent strain (ASFV-G) attenuated virulence and protected against challenge with parental strains (48). The vaccine candidate ASFV-G-ΔI177L can effectively protect against a circulating Vietnamese field strain in both Vietnamese and European pigs, but the efficacy of this live attenuated vaccine at a larger scale remains unknown (49). Given the biological significance of the ASFV MGF360/530 gene family in inhibiting the IFN response, deleting genes that inhibit IFN functions is a promising approach to generate attenuated ASFV candidate vaccine strains (50). In this study, zero animals infected with ASFV-Δ10L (10 HAD_50_) died, and the viral loads in the tissue, blood, and swabs were lower than in animals infected with parental ASFV-WT. The present study demonstrated, for the first time, that ASFV-Δ10L is a virulence factor that could be used as a vaccine candidate against ASFV infection.

In conclusion, our study showed that ASFV MGF-360-10L could inhibit the IFN-β-stimulated STAT1/2 pathway by targeting JAK1 and promoting the K48 ubiquitination of JAK1 through the E3 ubiquitin ligase HERC5. We further demonstrated that the lethality and the viral loads after infection with ASFV-Δ10L were significantly lower than that of the parental ASFV-WT, indicating that MGF-360-10L is a new ASFV virulence factor. We also explored the specific mechanisms by which MGF-360-10L affects the STAT1/2 innate immune pathway to provide a strong theoretical basis for further research and developing ASFV vaccines.

## MATERIALS AND METHODS

### Animal experiments

Specific pathogen-free swine were used to detect the virulence of the parental ASFV-WT virus and ASFV-Δ10L. Six control and six experimental animals were injected with 10 HAD_50_ of ASFV-WT and ASFV-Δ10L, respectively. Daily temperature monitoring and collection of blood samples and fecal, oral, and nasal swabs were performed for 19 days. Increased body temperature and other clinical symptoms of the ASFV-inoculated pigs were scored according to a clinical scoring system (51).

### Cell culture and virus infection

PAMs were prepared by bronchoalveolar lavage, and porcine bone marrow-derived macrophages (BMDMs) were prepared from bone marrow according to previously published methods (52, 53) and grown in Roswell Park Memorial Institute 1640 (Solarbio Life Science, China) medium containing 1% penicillin-streptomycin-gentamicin solution (Solarbio Life Science, China) and 10% porcine serum. HEK293T cells were cultured in Dulbecco’s modified Eagle’s medium (DMEM) (Invitrogen, Waltham, MA, USA) supplemented with 1% penicillin-streptomycin-gentamicin solution and 10% fetal bovine serum. All cells were cultured at 37°C under 5% CO_2_ atmosphere saturated with water vapor. ASFV-WT was propagated on PAMs as previously described (54) and stored at −80°C.

### Plasmid construction

The STAT1/2 and IRF1 promoter luciferase reporter plasmids and mammalian expression plasmids for HA-tagged Ub, K48, K63, and Myc-JAK1 used in the present study were described previously (55). The mammalian expression plasmids for Myc-HERC5 and Myc-HERC5 (C994A) were constructed using standard molecular biology techniques. To construct Flag-MGF-360-10L, a DNA fragment was amplified by PCR from the cDNA of ASFV-WT, which contains full-length MGF-360-10L and subcloned into the pCMV-3 × FLAG vector.

### ASFV-Δ10L and ASFV-10L-GFP construction

Homologous recombination was used to construct an MGF-360-10L-deficient virus, denoted ASFV-Δ10L, and a 3′-terminally eGFP-tagged virus, denoted ASFV-10L-GFP. The recombinant plasmids were first constructed according to a previous publication (56), with pPL101 or pUC57 used as the backbone. Primers were designed to amplify MGF-360-10L using the ASFV-WT strain as a template. The left and right homology arms of MGF-360-10L, located from nucleotides (nt) 27,543 to 26,482 and 25,216 to 24,164, respectively, were also amplified by PCR, as well as the p72 promoter sequence and eGFP. These DNA elements were combined using a multi-fragment homologous recombinase (TYSW-CL08020), and the construction was verified by Sanger sequencing. ASFV-10L-GFP was constructed similarly to ASFV-Δ10L, except that an eGFP tag was added to the 3′-terminus of MGF-360-10L. BMDMs were transfected with the homologous recombinant plasmids pPL101-Δ10L-eGFP and pUC57-10L-eGFP using jetPRIME *in vitro* transfection reagent (Polyplus, France), and cells were subsequently infected with 0.01 MOI of ASFV-WT. After culturing for 24 h, single fluorescent cells were selected under a fluorescence microscope and were frozen at −80°C prior to downstream analysis. Single fluorescent cells were inoculated into 96-well plates with BMDMs to continue culturing and were denoted as F1. The purified ASFV-Δ10L gene knockout virus was obtained by limiting dilution assay, amplification, and culturing to about F10. PCR primers were designed to detect the purity of the ASFV-Δ10L viral strain.

### Antibodies and reagents

Rabbit anti-p72 was raised against recombinant ASFV p72 protein. Polyclonal rabbit anti-JAK1 (29261), phospho-JAK1 (Tyr1034/1035) (3331S), JAK2 (3230), TYK2 (9312S), phospho-TYK2 (Tyr1054/1055) (9321S), STAT1 (14994), phospho-STAT1 (Tyr701) (9167), STAT2 (D9J7L), and phospho-STAT2 (Y690) (AP0284) were purchased from Cell Signaling Technology (USA). Polyclonal rabbit anti-HERC5 was purchased from Boster Bio. Monoclonal mouse anti-HA (H3663), β-actin (A5441), Myc (SAB1305535), and Flag (F3040) were purchased from Sigma-Aldrich (USA). Monoclonal mouse anti-GFP (sc-9996) was purchased from Santa Cruz Biotechnology (USA). Alexa Fluor-488-conjugated goat anti-mouse IgG (H + L) and Alexa Fluor 594-conjugated goat anti-rabbit IgG (H + L) antibodies were purchased from Cell Signaling Technology. 3-Methyladenine (3-MA) (Catalogue #189490) and MG132 (M8699) were purchased from Sigma-Aldrich.

### Transfection and reporter gene assays

HEK293T cells were digested with trypsin, resuspended in DMEM containing 10% fetal bovine serum, and seeded in 24-well plates. Once the cells reached a 40%–60% confluency, the standard calcium phosphate precipitation method was used for cell transfection. Cells were co-transfected with empty and Flag-MGF-360-10L plasmids and STAT1/2 and IRF1 reporter plasmids and stimulated with IFN-β or IFN-γ. Luciferase assays were performed using a dual-specific luciferase assay kit (Promega, Madison, Wisconsin, USA), and firefly luciferase activity was normalized to Renilla luciferase activity.

### Confocal microscopy

HEK293T cells were transfected with Flag-MGF-360-10L and Myc-JAK1 expression plasmids and then cultured for 24 h at 37°C under 5% CO_2_ atmosphere saturated with water vapor. The cells were treated with IFN-β for 2 h or with DMEM as the control. The supernatant was discarded, and the residual medium was removed by washing with phosphate-buffered saline before the cells were fixed with 4% paraformaldehyde pre-cooled to 4°C for 30 min at room temperature. Cells were permeabilized with 0.3% Triton-X100 for 10 min and blocked with 5% BSA for 1 h at room temperature. The permeabilized cells were incubated with monoclonal rabbit anti-Flag and monoclonal mouse anti-Myc overnight at 4°C and then incubated with Alexa Fluor 488 anti-rabbit (Catalog 4408S) and Alexa Fluor 594 anti-mouse (8890S) (Cell Signaling Technology, Danvers, MA, USA) for 1 h at room temperature in the dark. Cell nuclei were stained with 4′, 6′-diamidino-2-phenylindole for 10 min. The cells were visualized using a Leica SP2 confocal microscopy system (Leica Microsystems, Wizla, Germany).

### siRNA knockdown

siRNAs corresponding to the porcine HERC5 target sequence were purchased from (Sangon Biotech, China). PAMs were transfected with negative control RNAi and HERC5-RNAi using jetPRIME reagent for 48 h. The cells were infected with 0.05 MOI of ASFV-10L-GFP for 0, 12, 24, and 48 h. Protein expression was detected by Western blotting using the indicated antibodies.

### qPCR

The expression of *ISG15*, *IRF9*, *GBP1*, and other indicated genes was determined by real-time RT-PCR using the SYBR Green detection system. HEK293T cells were transfected with Flag-MGF-360-10L or empty vector for 24 h and then either treated with IFN-β or IFN-γ for 2 h, respectively, or with blank BMDM as the control. PAMs were infected with 0.01 MOI ASFV for 24 h, then treated with IFN-β for 4 h. Total RNA was extracted from HEK293T and PAMs using TRIzol reagent according to the manufacturer’s instructions (Sigma-Aldrich, USA), and cDNA was generated by reverse transcription using the Prime Script RT reagent kit (Takara Bio, Japan). To determine the relative mRNA abundance, qPCR was performed using the TB Green Fast qPCR Mix (Takara Bio, Japan). Data were analyzed using Statistic 17.0 software. The qPCR primer sequences used are listed in Table 1. Human and porcine GAPDH were used as reference genes.

**TABLE 1:** The primers sequence in this study

Primers	Sequences (5'–3')
Porcine GAPDH-F	ACATGGCCTCCAAGGAGTAAGA
Porcine GAPDH-F	GATCGAGTTGGGGCTGTGACT
Porcine ISG15-F	CCTGTTGATGGTGCAAAGCT
Porcine ISG15-R	TGCACATAGGCTTGAGGTCA
Porcine ISG54-F	CTGGCAAAGAGCCCTAAGGA
Porcine ISG54-R	CTCAGAGGGTCAATGGAATTCC
Porcine ISG56-F	TCAGAGGTGAGAAGGCTGGT
Porcine ISG56-R	GCTTCCTGCAAGTGTCCTTC
Porcine JAK1-F	CATTATGCAAGGCGAGCACC
Porcine JAK1-R	TCCTCAACACATTCGGGAGC
Porcine HERC5-F	GCCCTGTTTTGGGACAGAGT
Porcine HERC5-R	TCTGTGCTCTGATGGGGTCT
Porcine control-RNAi-F	UUCUCCGAACGUGUCACGUTT
Porcine control-RNAi-R	ACGUGACACGUUCGGAGAATT
Porcine HERC5-RNAi-F	CAGAAGGACAACUGGGAAATT
Porcine HERC5-RNAi-R	UUUCCCAGUUGUCCUUCUGTT
Human GAPDH-F	GAGTCAACGGATTTGGTCGT
Human GAPDH-F	GACAAGCTTCCCGTTCTCAG
Human Mx1-F	CAGGACATTTGAGACAATCGTG
Human Mx1-R	TCGAAACATCTGTGAAAGCAAG
Human ISG15-F	AGGACAGGGTCCCCCTTGCC
Human ISG15-R	CCTCCAGCCCGCTCACTTGC
Human ISG56-F	TCACAGGTCAAGGATAGTC
Human ISG56-R	CCACACTGTATTTGGTGTCTAGG
Human GBP1-F	TAGCAGACTTCTGTTCCTACATCT
Human GBP1-R	CCACTGCTGATGGCATTGACGT
Human JAK1-F	CTTTGCCCTGTATGACGAGAAC
Human JAK1-R	ACCTCATCCGGTAGTGGAGC
p72-F	CTGCTCATGGTATCAATCTTATCGA
p72-R	GATACCACAAGATC(AG)GCCGT

Next, to evaluate the genomic copies of ASFV, PAMs were infected with ASFV-WT or ASFV-Δ10L for 24 h, and total RNA was extracted using TRIzol reagent according to the manufacturer’s instructions (Sigma-Aldrich). cDNA was generated using the Prime Script RT reagent kit (Takara Bio). The conserved p72 gene segment of the ASFV genome was amplified using the primers listed in Table 1. A TaqMan probe (5′-[6-carboxy-fluorescein (FAM)]-ccacgggaggaataccaacccagtg-3′-[6-carboxy-tetramethyl-rhodamine (TAMRA)]) was obtained from Applied Biosystems (USA) and was designed based on the alignment of 54 available ASFV sequences and targeting the 3′ end of p72. Data analysis was performed using QuantStudio Design & Analysis Software (Applied Biosystems, USA).

### Co-immunoprecipitation and immunoblotting assay

For the transient transfection and co-immunoprecipitation experiments, HEK293T cells were transfected with the appropriate plasmids for 24 h. The cells were harvested and lysed in 1 mL of lysis buffer [20 mM Tris (pH = 7.5), 150 mM NaCl, 1% Triton, 1 mM EDTA, 10 mg/mL aprotinin, 10 mg/mL leupeptin, and 1 mM PMSF] at 4°C, followed by sonication and centrifugation. For each immunoprecipitation reaction, 0.4 mL of cell lysate was incubated with 0.5 mg of the indicated Ab or control IgG and 40 µL of protein G agarose beads (Santa Cruz Biotechnology, USA) at 4°C for 4 h. The beads were washed three times with 1 mL of lysis buffer containing 0.5 M NaCl. Samples were resolved using sodium dodecyl sulfate-polyacrylamide gel electrophoresis and transferred to a nitrocellulose membrane (Pall Corporation, Port Washington, NY, USA). The membranes were incubated with the indicated primary antibodies diluted in Tris-buffered saline (TBS) supplemented with 1% milk powder at room temperature. The membranes were washed three times with TBS supplemented with 1% Tween-80 and incubated with specific peroxidase-conjugated secondary antibodies at room temperature for 1 h. Bands were detected by chemiluminescence using ECL Prime reagent (Merck Millipore, USA). For the endogenous co-immunoprecipitation experiments, PAMs were infected with 0.01 MOI ASFV for the indicated times or with blank 1640 as the control.

### RNA-seq

PAMs were infected with ASFV-10L-GFP or ASFV-Δ10L for the indicated time, and total RNA was extracted from each sample using a TRIzol reagent. A NanoDrop 2000 spectrophotometer (Thermo Fisher Scientific, USA) was used to determine the concentration and purity of the RNA samples. An Agilent 2100 Bioanalyzer and a 2100 RNA Nano 6000 assay kit (Agilent Technologies, Santa Clara, California, USA) were used to evaluate the integrity of the RNA samples. After the QC procedures, RNA with poly-A tails was enriched using the TIANSeq mRNA Capture Kit (TIANGEN, China). The captured RNA was then used as the template for the TIANSeq Fast RNA Library Kit (Illumina, San Diego, California, USA). Briefly, transcriptome sequencing libraries were constructed by performing random RNA fragmentation, cDNA strand 1/strand 2 syntheses, end repair, A-tailing, ligation of sequencing adapters, size selection, and library PCR enrichment. The transcriptome libraries were analyzed to identify differentially expressed genes (DEGs) using topGO R packages based Kolmogorov-Smirnov test. Gene ontology terms with corrected *P* values <0.05 were considered significantly enriched among the DEGs, and “clusterProfiler” package was used to evaluate the enrichment of DEGs in KEGG pathways.

### Facility biosafety statement

All experiments with live ASFV were conducted within enhanced biosafety level 3 (P3) facilities at the Lanzhou Veterinary Research Institute of the Chinese Academy of Agricultural Sciences and were approved by the Ministry of Agriculture and Rural Affairs and the China National Accreditation Service for Conformity Assessment.

### Virus titration

The wild-type ASFV CN/GS/2018, ASFV-Δ10L, and ASFV-10L-GFP viruses were quantified using the HAD assay described previously (21). PAMs were infected with ASFV-WT, ASFV-10L, and ASFV-10L-GFP for the indicated times, and then the cells and culture medium were frozen and thawed at −80°C before dilution. PAMs were seeded in 96-well plates, and the samples were added to the plates and titrated in triplicate using 10-fold serial dilutions. Fresh suspensions of autologous swine erythrocytes were added to each sample. HAD was determined on day 7 post-inoculation, and 50% HAD doses (HAD_50_) were calculated using the method described by Reed and Muench (57). Growth curves were generated using GraphPad Prism (San Diego, CA, USA).

### Statistical analysis

All graphs were plotted using GraphPad Prism 5.0 (USA). Comparisons between different groups were analyzed using Statistic 17.0 (StatSoft, USA) using one-way analysis of variance. Differences were considered statistically significant at *P < 0.05, **P < 0.01, ***P < 0.001, and non-significant at *P* > 0.05.

## SUPPLEMENTAL MATERIAL

Fig. S1 mbio.00606-23-s0001.tiff ASFV MGF-360-10L negatively regulates IFN-β-triggered STAT1/2 signaling.

Fig. S2 mbio.00606-23-s0002.tif MGF-360-10L mediates JAK1 degradation via E3 ubiquitin ligase HERC5.

Supplemental legends mbio.00606-23-s0003.docx Legends to Fig. S1 and S2.

ASM does not own the copyrights to Supplemental Material that may be linked to, or accessed through, an article. The authors have granted ASM a non-exclusive, world-wide license to publish the Supplemental Material files. Please contact the corresponding author directly for reuse.
