# Supplementary figures and images for: African swine fever virus MGF-360-10L is a novel and crucial virulence factor that mediates ubiquitination and degradation of JAK1 by recruiting the E3 ubiquitin ligase HERC5
Source: mBio. 2023 Jul 7;14(4):e00606-23. doi: 10.1128/mbio.00606-23 (PMC10470787; doi:10.1128/mbio.00606-23)

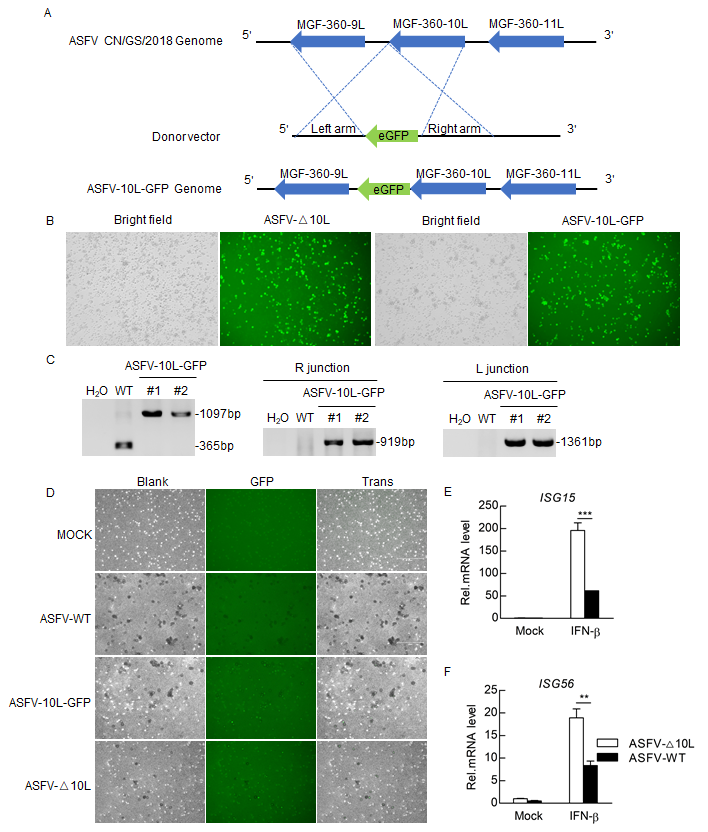

Supplement: Fig. S1 — ASFV MGF-360-10L negatively regulates IFN-β-triggered STAT1/2 signaling. [file mbio.00606-23-s0001.tiff]

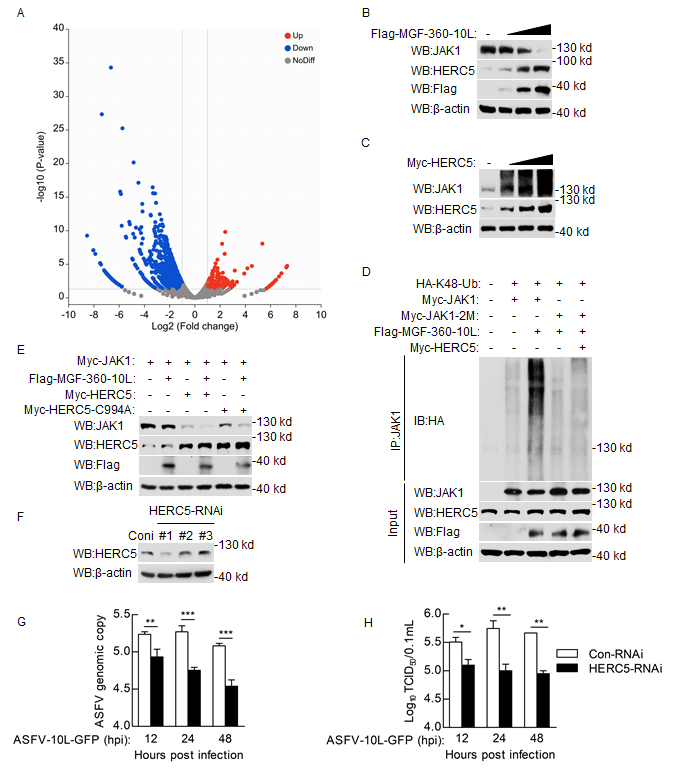

Supplement: Fig. S2 — MGF-360-10L mediates JAK1 degradation via E3 ubiquitin ligase HERC5. [file mbio.00606-23-s0002.tif]
