# Supplementary material for: African swine fever virus MGF-360-10L is a novel and crucial virulence factor that mediates ubiquitination and degradation of JAK1 by recruiting the E3 ubiquitin ligase HERC5
Source: mBio. 2023 Jul 7;14(4):e00606-23. doi: 10.1128/mbio.00606-23 (PMC10470787; doi:10.1128/mbio.00606-23)
Supplement: Supplemental legends — Legends to Fig. S1 and S2. [file mbio.00606-23-s0003.docx]

**Supplementary Figure 1.** ASFV MGF-360-10L negatively regulates IFN-β-triggered STAT1/2 signaling. (A) Diagram indicating the position of the MGF-10L-GFP open reading frame in the ASFV CN/GS/2018 genome. (B) Successful recombination was confirmed using a fluorescence microscope. (C) The absence of parental CN/GS/2018 was confirmed using PCR. (D) PAMs were infected with ASFV-10L-GFP, ASFV-WT or ASFV-Δ10L, and then swine erythrocytes were added to develop hemadsorption. (E-F) PAMs were infected with ASFV-WT or ASFV-Δ10L for 24 h, and then treated with IFN-β (0.7 μg/ml) for 4 h before *ISG15* (E) and *ISG56* (F) were detected by qPCR. Data represent the mean ± SD, n =3. **P*＜0.05, ***P*＜0.01, ****P*＜0.001.

**Supplementary Figure 2.** MGF-360-10L mediates JAK1 degradation via E3 ubiquitin ligase HERC5. (A) PAMs were infected with ASFV-Δ10L or ASFV-10L-GFP (MOI: 0.1) for 24 h. Proteins upregulated in infected cells are shown in red, while downregulated proteins are shown in blue. (B-C) HEK293T cells were transfected with Flag-MGF-360-10L (0, 0.5, 1, and 2.0 μg) (B), Myc-HERC5 (0, 0.5, 1, and 2.0 μg) (C), then cell lysates were analyzed by Western blotting using the indicated antibodies. (D) HEK293T cells were transfected with Flag-MGF-360-10L, Myc-HERC5, HA-K48, and Myc-JAK1 or Myc-JAK1 mutants, co-immunoprecipitation, and Western blotting analyses were performed using the indicated antibodies. (E) HEK293T cells were transfected with Myc-JAK1, Flag-MGF-360-10L, Myc-HERC5 or mutants expression plasmids for 24 h, and cell lysates were analyzed by Western blotting. (F) PAMs were transfected with Con-RNAi or HERC5-RNAi for 48 h, and the expression of HERC5 was detected by Western blotting. (G-H) PAMs were transfected with Con-RNAi or HERC5-RNAi for 48 h, and the number of genome copies and HAD_50_ were detected. Data represent the mean ± SD, n =3. **P*＜0.05, ***P*＜0.01, ****P*＜0.001.
